# Differences of body composition and physical strength among Japanese and Thai older adults living in Chiang Mai, Thailand: an inter-ethnic cross-sectional study

**DOI:** 10.1186/s12199-021-01017-0

**Published:** 2021-09-29

**Authors:** Takeshi Yoda, Bumnet Saengrut, Kensaku Miyamoto, Rujee Rattanasathien, Tatsuya Saito, Yasuko Ishimoto, Kanlaya Chunjai, Rujirat Pudwan, Kawin Sirimuengmoon, Hironobu Katsuyama

**Affiliations:** 1 Department of Health and Sports Science, Kawasaki University of Medical Welfare, 288 Matsushima, Kurashiki City, Okayama, 701-0193 Japan; 2 Department of Public Health, Kawasaki Medical School, Kurashiki, Japan; 3 Nursing Service Department, Maharaj Nakorn Chiang Mai Hospital, Faculty of Medicine, Chiang Mai University, Chiang Mai, Thailand; 4 Faculty of Education, Kagawa University, Takamatsu, Japan; 5 Japan Institute of Sports Sciences, Tokyo, Japan; 6 Faculty of Associated Medical Sciences, Chiang Mai University, Chiang Mai, Thailand

**Keywords:** Body composition, Skeletal muscle mass index, Sarcopenia, Japanese, Thai, Older adults

## Abstract

**Background:**

The number of adults aged over 65 years is rapidly increasing in several Southeast Asian countries. Muscle mass decreases with age, leading to sarcopenia. The primary objective of this study was to determine whether differences exist in the body composition and physical strength, according to ethnicity, among community-dwelling Japanese and Thai older adults living in Chiang Mai Province, Thailand.

**Methods:**

A survey was conducted in February and March 2019. Japanese and Thai adults aged ≥ 60 years living in Chiang Mai Province were recruited through community clubs. Participants completed a self-administered questionnaire that enabled collection of data on age, sex, educational background, marital status, annual income, current medical conditions, smoking and alcohol consumption, and exercise habits. Measurements were collected on height, weight, body composition, blood pressure, hand grip, and walking speed for 6 m. Body composition was measured using a standing-posture 8-electrode multifrequency bioimpedance analysis analyzer. Hand grip of each hand was measured with the patient in the standing position using a digital grip dynamometer. Multivariable logistic regression was used to determine factors associated with skeletal muscle mass index (SMI).

**Results:**

Of the total 119 participants, 47 were Japanese (26 men, 21 women) and 72 were Thai (16 men, 56 women). The prevalence of a low SMI was 3/26 (12%), 1/21 (5%), 6/16 (38%), and 5/56 (9%) among Japanese men, Japanese women, Thai men, and Thai women, respectively. The prevalence of low muscle strength was 2/26 (8%), 2/21 (10%), 3/16 (19%), and 13/56 (23%) among Japanese men, Japanese women, Thai men, and Thai women, respectively. There were significant differences between ethnic groups in body mass index for both sexes, percentage body fat in women, SMI in men, and average grip strength in men. Ethnic group, sex, age, and body mass index were independent predictors of SMI.

**Conclusions:**

Ethnicity had a clinically important effect on body composition and physical strength among older Japanese and Thai adults living in a similar environment.

## Background

In Asian countries, the population is aging. The number of adults aged over 65 years is rapidly increasing in Japan and several other Southeast Asian countries [1]. Muscle mass decreases with age, leading to sarcopenia [2]. According to the diagnostic criteria defined by the Asian Working Group for Sarcopenia (AWGS), sarcopenia is diagnosed when there is low muscle mass (defined as a skeletal muscle mass index [SMI] < 7 kg/m^2^ in males and < 5.7 kg/m^2^ in females), with either low muscle strength (defined as handgrip strength < 26 kg in males and < 18 kg in females) or low physical performance (defined as 6-m gait speed ≤ 0.8 m/s), or both [3]. Sarcopenia is associated with poor quality of life [4, 5] and future risk of physical disability [6–8]. The age-related muscle mass loss is also associated with an increased risk of incident disability [9] and hospitalization [10], increased all-cause mortality [11, 12], and higher healthcare costs [13] in the older adults. Therefore, it is important to prevent sarcopenia older adults. Epidemiological studies from Asian countries, which used the AWGS 2014 criteria, found a prevalence of sarcopenia ranging from 5.5 to 25.7% [14]. The prevalence of sarcopenia has been reported to differ by race among inpatients and older nursing home residents [15–17], but only few studies have assessed this aspect in community-dwelling older adults. We conducted a study to assess on the quality of life and mental health status of Japanese older adults living in Chiang Mai, Thailand [18]. All participants were community dwellers―not inpatients or nursing home residents, and they were born in Japan. In addition, almost all of them formerly lived in Japan; they emigrated to Chiang Mai after retirement. We assessed the body composition, using bioelectrical impedance analysis (BIA), and physical strength, with the hand grip and walking speed tests, for both Thai and Japanese older adults living in Chiang Mai Province, Thailand. Although the Japanese immigrants did not live in Chiang Mai all through their lives, their living environment after migration, including the temperature and humidity they were exposed to, was the same as those experienced by the local Thai older adults. It was therefore important to compare the body composition and physical strength of these groups to eliminate confounding environmental factors. The primary objective of this study was to determine the differences in body composition and physical strength, according to ethnicity, among community-dwelling Japanese and Thai older adults living in the same environment.

We hypothesized that Thai older adults had better body composition and physical strength than Japanese older adults. The reason for this hypothesis was that many of the older people living in the suburbs of Chiang Mai used to be or are still partially engaged in agricultural work, so they are probably more physically active on a daily basis than Japanese retirees. In addition, they probably eat traditional Thai food, mainly rice, on a daily basis, which may affect their body composition from a nutritional point of view.

## Methods

### Target population

We conducted a survey in February and March 2019. The study population included Japanese and Thai older adults. For Japanese, the study population included long-stay Japanese older adults living in Chiang Mai Province who were defined as those: (1) at least 60 years old, (2) retired from work in Japan with a desire to stay in Thailand, and (3) living in Thailand for at least 3 months at the time of the study [18]. Chiang Mai, in northern Thailand, was chosen as the study site because the City of Chiang Mai is a popular location for Japanese long-stayers [19], and the Chiang Mai Province is one of five pilot provinces for long-stay tourism in Thailand [20].

Participants were recruited from two clubs for Japanese older adults in Chiang Mai: the Chiang Mai Long-Stay Club for Japanese People and the Chiang Mai Expatriates Club for Japanese People. As these clubs hold monthly meetings, we visited each club during one of its meetings in February 2019. At the meetings, we explained the study purpose and research details; after obtaining consent, we measured some items and administered a self-reported questionnaire to collect sociodemographic data. The survey was conducted by the Japanese researchers group themselves.

The same methods were used for Thai older adults living in Chiang Mai Province. Thai participants were a convenience sample of members of two community clubs for older adults, registered with the Nursing Department, Chiang Mai University. These clubs were selected because they had been surveyed in previous study on community health. The measurement methods were same as those used in the Japanese participants. The survey was conducted by the Thai researchers group themselves.

### Measurement items

The measurement items were as follows: height, weight, body composition, blood pressure, hand grip, and walking speed for 6 m. Body composition was measured using a standing-posture 8-electrode multifrequency BIA analyzer (MC-780-A-N, Tanita, Tokyo, Japan). Skeletal muscle mass is sometimes measured using dual-energy X-ray absorptiometry (DXA) methods for sarcopenia diagnosis (1), but DXA is expensive, has limited portability and requires radiation exposure. Compared with DXA, BIA is inexpensive, easy to use, portable, and requires no radiation exposure [21–23]. However, a large variety of models have been commercially supplied by different companies; for most consumer products, the equations used to estimate appendicular skeletal muscle mass (ALM) are not disclosed [24]. We chose to use the Tanita MC-780-A-N because it had already been validated for estimating ALM by Yamada et al. [25] using a theoretical, age-independent equation. Blood pressure was measured with an automated sphygmomanometer (HEM-7130-HP, Omron, Kyoto, Japan). Each participant’s blood pressure was measured twice, once on each arm. Hand grip was measured using digital grip dynamometer (GRIP-D T.K.K.5401, Takei Scientific Instruments, Niigata, Japan). Hand grip was also measured twice on the left and right with the participant in a standing position.

Participants completed a self-administrated questionnaire that enabled collection of data on age, sex, educational background, marital status, annual income, current medical conditions, smoking and alcohol consumption, and exercise habits.

### Data analysis

Descriptive statistics were used to compare ethnic differences between Japanese and Thai participants. We used cut-off of SMI < 7 kg/m^2^ in males and < 5.7 kg/m^2^ in females to detect possible sarcopenia combined with either low muscle strength (defined as handgrip strength < 26 kg in males and < 18 kg in females) or low physical performance (defined as 6-m gait speed ≤ 0.8 m/s) according to the AWGS criteria. Measurement items were averaged and compared by sex and ethnic group. Questionnaire items were also categorized by sex and ethnic group. The mean and standard deviation were calculated for continuous variables. Two-sided *t* tests were used to compare continuous variables and Chi-square tests were used to compare categorical variables according to sex and ethnicity. *P* values < 0.05 were considered statistically significant. Multiple regression analysis was used to determine factors associated with the SMI. All statistical analyses were performed using JMP 14.0 (SAS Institute Inc., Cary, NC, USA).

### Ethics approval and informed consent

The study protocol was approved by the ethical committees of Kawasaki University of Medical Welfare (Approval number: 18-102) and Chiang Mai University (Approval number: NUR-2562-06120). All participants provided written informed consent to participate in the study.

## Results

There were a total of 119 participants, including 47 Japanese participants (26 men, 21 women) and 72 Thai participants (16 men, 56 women). The average age of the Japanese participants was 71.1 years (standard deviation, 4.89 years), and the average age of the Thai participants was 68.8 years (standard deviation, 5.61 years). Other sociodemographic characteristics are shown in Table 1.

**Table 1:** Socio-demographic characteristics

		Male			Female		
		Japanese	Thai	p*	Japanese	Thai	p*
Average age	Mean (SD)	71.4 (5.4)	69.9 (6.9)	0.42	68.8 (8.5)	67.2 (6.1)	0.39
Marital status	Single	6	4	0.93	2	28	< 0.01
	Married	17	12		16	28	
Education	Primary school	0	4	< 0.01	0	32	< 0.01
	Junior high school	0	1		1	6	
	Senior high school	3	2		6	6	
	College	0	3		8	3	
	University	20	5		3	8	
	Graduate school	0	1		0	1	
Chronic diseases	None	3	7	0.02	11	11	< 0.01
	1+	23	9		10	45	
Drinking alcohol	No	5	16	< 0.01	12	56	< 0.01
	Yes	18	0		6	0	
Smoking	Non-smoker	21	16	-	16	55	0.48
	Smoker	0	0		0	1	
Daily exercise	Yes	17	12	0.93	13	53	0.01
	Never	6	4		5	3	

*SD* standard deviation

*Chi-square test (exclude average age). Average age were used by *t* test (two-sided)

Table 2 shows the differences in the measurement items according to ethnicity and sex. There were significant differences between ethnic groups in height in women (mean, 156.0 cm and 151.9 cm in Japanese and Thai women, respectively, *p* < 0.01); body mass index (BMI) for both sexes (mean, 24.2 kg/m^2^ and 22.5 kg/m^2^ in Japanese and Thai men, respectively, *p* = 0.04; mean, 22.9 kg/m^2^ and 25.0 kg/m^2^ in Japanese and Thai women, respectively, *p* = 0.04); systolic blood pressure in men (mean, 147.3 mmHg and 129.1 mmHg in Japanese and Thai men, respectively, *p* = 0.01); diastolic blood pressure for both sexes (mean, 85.8 mmHg and 74.3 mmHg in Japanese and Thai men, respectively, *p* < 0.01; mean, 78.5 mmHg and 72.7 mmHg in Japanese and Thai women, respectively, *p* < 0.01); and percentage body fat in women (mean, 32.9% and 37.6% in Japanese and Thai women, respectively, *p* = 0.02).

**Table 2:** Ethnic differences for average measurement variables by gender

		Male			Female		
		Japanese	Thai		Japanese	Thai	
		*N* = 26	*N* = 16	p*	*N* = 21	*N* = 56	p*
Height	(cm)	167.4 (5.9)	165.6 (7.1)	0.37	156.0 (4.3)	151.9 (5.4)	< 0.01
Weight	(kg)	67.8 (7.6)	62.0 (11.8)	0.06	55.6 (8.9)	57.6 (9.4)	0.38
BMI		24.2 (2.2)	22.5 (3.2)	0.04	22.9 (4.0)	25.0 (4.0)	0.04
SBP	(mmHg)	147.3 (23.2)	129.1 (17.1)	0.01	136.3 (13.0)	131.4 (14.5)	0.21
DBP	(mmHg)	85.8 (9.9)	74.3 (9.8)	< 0.01	78.5 (8.1)	72.7 (7.2)	< 0.01
%BF	(%)	24.9 (4.4)	22.4 (8.0)	0.19	32.9 (6.1)	37.6 (8.2)	0.02
SMI	(kg/m^2^)	7.9 (0.7)	7.3 (0.9)	0.03	6.6 (0.7)	6.4 (0.5)	0.21
Hand grip	(kg)	35.1 (5.6)	30.8 (7.5)	0.04	22.6 (4.8)	21.5 (4.2)	0.37
Walking speed	(m/s)	1.3 (0.2)	1.9 (0.4)	< 0.01	1.4 (0.2)	1.5 (0.3)	0.07

*BMI* body mass index, *SBP* systolic blood pressure, *DBP* diastolic blood pressure, *%BF* percentage of body fat, *SMI* skeletal muscle mass index

*t test (two-sided)

**Fig. 1:**
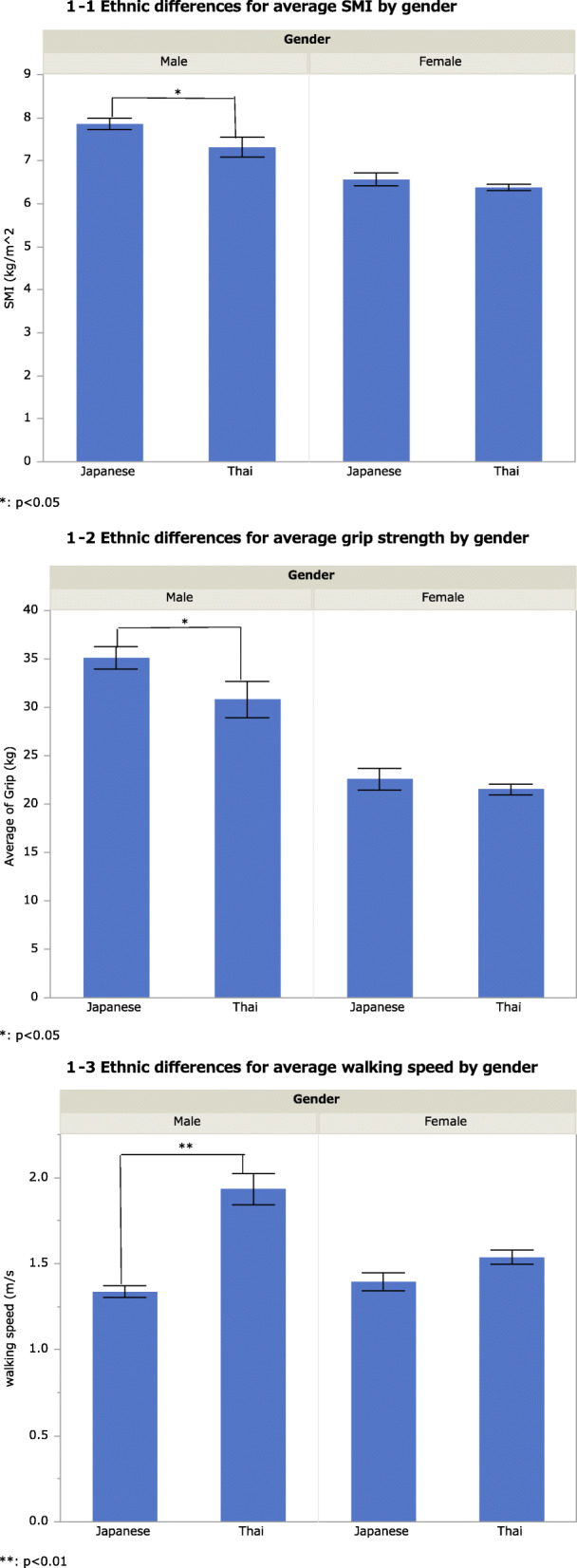
Ethnic differences for average SMI (1-1) (*p < 0.05), average grip strength (1-2) (*p < 0.05), and average walking speed (1-3) (**p < 0.01) by gender. Each error bar was shown as standard error

Figure 1 shows the average SMI, average grip strength, and walking speed. In men, there were significant differences between ethnic groups in the SMI (mean, 7.85 kg/m^2^ and 7.31 kg/m^2^ in Japanese and Thai men, respectively, *p* = 0.03), average grip strength (mean, 35.1 kg and 30.7 kg in Japanese and Thai men, respectively, *p* = 0.04), and average walking speed (mean, 1.3 m/s and 1.9 m/s in Japanese and Thai men, respectively, *p* < 0.01).

Low SMI was seen in 3/26 (12%), 1/21 (5%), 6/16 (38%), and 5/56 (9%) Japanese men, Japanese women, Thai men, and Thai women, respectively. The prevalence of low muscle strength was 2/26 (8%), 2/21 (10%), 3/16 (19%), and 13/56 (23%) among Japanese men, Japanese women, Thai men, and Thai women, respectively.

To investigate the factors associated with SMI, we conducted a multiple regression analysis with SMI as the dependent variable and sex, age, BMI, ethnic group, and other lifestyle habits as independent variables (Table 3). Ethnic group, sex, age, and BMI were significant predictors of the SMI. However, chronic diseases and lifestyle habits (exercise and alcohol consumption) were not significant predictors of the SMI.

**Table 3:** Multiple regression analysis of various factors in relation to SMI

Variables		*β*	*SE*	*p*
Ethnic group	(0:Japanese, 1:Thai)	−0.162	0.08	0.04*
Gender	(0:Male, 1:Female)	0.589	0.063	< 0.01*
Age		−0.025	0.009	< 0.01*
BMI		0.092	0.015	< 0.01*
Chronic diseases	(0:None, 1:1+)	−0.029	0.061	0.63
Exercise	(0:Never, 1:Yes)	0.035	0.077	0.65
Alcohol	(0:No, 1:Yes)	0.116	0.094	0.22
*R*^2^		0.652		< 0.01*
Adjusted *R*^2^		0.628		

*SMI* skeletal muscle mass index, *BMI* body mass index, *SE* standard error

*p < 0.05

## Discussion

We identified several participants with possible sarcopenia (low SMI) and low muscle strength (grip). Even though gait speed among the older Thai participants was significantly more than that of the Japanese participants, the other items measured were lower in Thai participants than in Japanese participants. We initially hypothesized that Thai older adults would have a better SMI and physical strength than Japanese older adults. However, the Japanese older adults scored better in both SMI and physical strength than the Thai older adults. Possible explanations for our results include the following: (1) Japanese older adults may be more active than the Thai older adults. This can be inferred from our previous study, which found that Japanese older adults in Chiang Mai engaged in sports activities in their leisure time [18]; (2) agricultural activities may not require as much physical strength as expected. Although these activities used to be completely manual, they are now mostly automated. The physical activity involved in farming will likely decrease even more with increased mechanization. However, a Korean study on the relationship between occupation and SMI found that those in agribusiness had a higher SMI and lower body fat mass than white-collar, blue-collar, and pink-collar workers [26]. Although the results of this study is contrary to our current result, it is worth investigating in future studies because our sample size was relatively small; (3) influenced by differences in eating habits. Japanese older adults live in an environment where Thai and other national cuisines are relatively accessible, in addition to the Japanese food they ate while living in Japan. In other words, they have a rich diversity of food. On the other hand, the Thai older people, although they live in the suburbs of Chiang Mai, have little choice to take them to the city center because public transportation is not well developed. As a result, their diet consists only of rice dishes, suggesting that they may be nutritionally unbalanced. In addition, Japanese older people diet habits are more likely seafood since Japan is an island country, while Thai people whose dietary pattern is more traditional rice and vegetable based since they live in Chiang Mai which is a mountainous area. However, we cannot actually conclude this because we have not done any nutritional research. Further research is needed; (4) differences in attitudes toward health. The educational backgrounds were different, and if people have less opportunity to learn about how to stay healthy in old age, they will not be as concerned about it. However, since Thailand is one of the countries where the idea and behavior of active aging is quite advanced, it is hard to imagine that the elderly are completely unaware of this idea and behavior [27].

On the other hand, percentage body fat was significantly higher among Thai women than among Japanese women. Physical function has been reported to be markedly decreased among older adults with low muscle mass and visceral fat obesity, compared to older adults with low muscle mass alone or obesity alone [28, 29]. Compared to simple obesity, sarcopenic obesity has been reported to cause an increase in the risk of restricted instrumental activities of daily living, frailty, falls, and walking disorders [28, 30, 31]. We independently examined obesity-related factors such as body weight, BMI, and percentage of body fat, as well as sarcopenia-related factors such as SMI, hand grip, and walking speed; however, we did not assess the correlations between these factors because this study did not aim to address sarcopenic obesity. We plan to assess the correlates of sarcopenic obesity in future research.

The multivariable logistic regression revealed that ethnic differences were significantly associated with SMI, whereas chronic diseases and lifestyle habits such as exercise and alcohol consumption were not. Previous studies have found that ethnicity is a major determinant of SMI and sarcopenia-related diseases and injuries [31–35]. Our study results suggest that there were ethnic differences among the Japanese and Thai older adults who participated in the study, even though their environmental conditions were similar. The results of the multivariable logistic regression also suggest that gender, age, and BMI are major determinants of SMI. Other studies have also shown that these are important factors that influence SMI [36–39]. Owing to our small sample size, it is difficult to generalize our research results regarding the determinants of SMI. However, as aforementioned, our results are supported by previous studies. Also, these results may possibly be influenced by environmental factors prior to migration. This is one of the issues to be discussed in the future.

There are some limitations to our study. First, the study sample was not randomly selected. The study population was small, and it was not feasible to conduct a household survey; thus, the approach of recruiting participants from clubs was considered a practical way to obtain a suitable study sample, although the possibility of selection bias cannot be ignored. In addition, the sample size was relatively small; thus, some important differences may not have been statistically significant. Second, there were gender differences between Japanese and Thai participants. Ideally, the number and gender ratio of Japanese and Thai participants should be equal. However, due to various restrictions, the number of participants and the ratio of male and female participants in this study were skewed. Third, since the study design was cross-sectional, we cannot draw any conclusions on the causality of observed associations between ethnicity and the other measured items. Fourth, some participants could not understand the instructions on how to walk at a normal speed and how to use the grip dynamometer. This might have affected the results, even though the influence is likely to have been small.

Despite these limitations, our research results revealed that there existed potential differences in SMI and grip strength between Japanese and Thai older adults living in the same environment. Our findings suggest that sarcopenia prevention strategies should take ethnicity into account.

## Conclusions

Our comparative study on body composition and physical strength between Japanese and Thai older adults suggests that ethnic differences may have a major role in this regard despite living environments being similar.

## Data Availability

The data presented in this study are available on request from the corresponding author (T.Y.). The data are not publicly available due to privacy concerns.

## Abbreviations

ALM: Appendicular skeletal muscle mass
AWGS: Asian Working Group for Sarcopenia
BIA: Bioelectrical impedance analysis
BMI: Body mass index
DXA: Dual-energy X-ray absorptiometry
SMI: Skeletal muscle mass index
